# Breast cancer pathology and stage are better predicted by risk stratification models that include mammographic density and common genetic variants

**DOI:** 10.1007/s10549-019-05210-2

**Published:** 2019-04-02

**Authors:** D. Gareth R. Evans, Elaine F. Harkness, Adam R. Brentnall, Elke M. van Veen, Susan M. Astley, Helen Byers, Sarah Sampson, Jake Southworth, Paula Stavrinos, Sacha J. Howell, Anthony J. Maxwell, Anthony Howell, William G. Newman, Jack Cuzick

**Affiliations:** 1 Division of Evolution and Genomic Sciences, Faculty of Biology, Medicine and Health, University of Manchester, MAHSC, Manchester, UK; 2 Prevention Breast Cancer Unit and Nightingale Breast Screening Centre, Manchester University NHS Foundation Trust (South), Manchester, UK; 3 Division of Informatics, Imaging and Data Sciences, School of Health Sciences, Faculty of Biology, Medicine and Health, University of Manchester, Manchester, UK; 4 Manchester Academic Health Science Centre, University of Manchester, Manchester, UK; 5 Centre for Cancer Prevention, Wolfson Institute of Preventive Medicine, Charterhouse Square, Barts and The London, Queen Mary University of London, London, UK; 6 The Christie NHS Foundation Trust, Manchester, UK; 7 Manchester Centre for Genomic Medicine, Manchester University NHS Foundation Trust (Central), Manchester, UK; 8 Manchester Breast Centre, Manchester Cancer Research Centre, University of Manchester, Manchester, UK; 9 NIHR Manchester Biomedical Research Centre, Cancer Prevention Early Detection Theme, The Christie NHS Foundation Trust, Manchester, UK; 10 Department of Genomic Medicine, Manchester Academic Health Sciences Centre (MAHSC), St Mary’s Hospital, University of Manchester, Manchester, M13 9WL UK

**Keywords:** SNPs, Polygenic risk score, Breast cancer, Mammographic density, Pathology, Early detection

## Abstract

**Purpose:**

To improve breast cancer risk stratification to enable more targeted early detection/prevention strategies that will better balance risks and benefits of population screening programmes.

**Methods:**

9362 of 57,902 women in the Predicting-Risk-Of-Cancer-At-Screening (PROCAS) study who were unaffected by breast cancer at study entry and provided DNA for a polygenic risk score (PRS). The PRS was analysed alongside mammographic density (density-residual-DR) and standard risk factors (Tyrer-Cuzick-model) to assess future risk of breast cancer based on tumour stage receptor expression and pathology.

**Results:**

195 prospective incident breast cancers had a prediction based on TC/DR/PRS which was informative for subsequent breast cancer overall [IQ-OR 2.25 (95% CI 1.89–2.68)] with excellent calibration-(0.99). The model performed particularly well in predicting higher stage stage 2+ IQ-OR 2.69 (95% CI 2.02–3.60) and ER + BCs (IQ-OR 2.36 (95% CI 1.93–2.89)). DR was most predictive for HER2+ and stage 2+ cancers but did not discriminate as well between poor and extremely good prognosis BC as either Tyrer-Cuzick or PRS. In contrast, PRS gave the highest OR for incident stage 2+ cancers, [IQR-OR 1.79 (95% CI 1.30–2.46)].

**Conclusions:**

A combined approach using Tyrer-Cuzick/DR/PRS provides accurate risk stratification, particularly for poor prognosis cancers. This provides support for reducing the screening interval in high-risk women and increasing the screening interval in low-risk women defined by this model.

**Electronic supplementary material:**

The online version of this article (10.1007/s10549-019-05210-2) contains supplementary material, which is available to authorized users.

## Introduction

Breast cancer is the most commonly diagnosed cancer in women worldwide. In familial breast cancer just over half of all cases are explained by a known genetic component [1–3], predominantly pathogenic variants in *BRCA1* or *BRCA2* and single nucleotide polymorphisms (SNPs). SNPs account for more of the familial risk than all pathogenic variants in high or moderate-risk breast cancer genes [1–3]. SNPs also explain a large proportion of risk in women developing breast cancer in those without a family history. Therefore, at a population level, SNPs are more informative than screening for moderate and high-risk gene variants [4, 5]. Dependent on the genotype of susceptibility SNPs (i.e. 0, 1 or 2 risk alleles) and the individual odds ratios for each risk allele, a risk estimate can be derived to create a polygenic risk score (PRS) [6].

At present, breast cancer risk prediction models include classical risk factors, for example, current age, family history, age of menarche, first full-term pregnancy and menopause, body mass index, type and number of breast biopsies and use of hormone replacement therapy (HRT - dose, type and duration) [7, 8]. In addition, high mammographic density has been established as a well-delineated breast cancer risk factor and several studies show that incorporation of mammographic density improves the accuracy of risk prediction models [9, 10]. Recent studies consider the value of including SNP genotype data into risk prediction algorithms, with promising results [11–14].

We collected data on classical risk factors, mammographic density and 18 breast cancer susceptibility SNPs (SNP18) on women who did not have breast cancer at entry to PROCAS [14, 15]. Recently, we showed that by combining mammographic density and SNP18 data with the Tyrer-Cuzick (TC) risk prediction model v6, women aged 46–73 years could be accurately divided into four 10-year risk groups (< 2%-low, 2–3.49%-average, 3.5–4.99% above average and ≥ 5% moderate/high) [14–16]. However, improvements in risk stratification are required, to define groups more precisely and reduce the large numbers at average risk.

Here, we report on the incidence rates and pathology in risk groups defined by TC/mammographic density/SNP18 in the PROCAS study.

## Methods

A total of 57,902 women aged between 46 and 73 years from the Greater Manchester area were recruited to the PROCAS study between October 2009 and June 2015. Women were recruited at the time of attendance for mammographic screening in the National Health Service Breast Screening Programme (NHSBSP). Standard breast cancer risk factors were collected using self-completed two-page questionnaires. Saliva samples were collected from 9899 women after their initial study mammogram at drop-in days at several centres in Greater Manchester. In addition, samples were specifically collected from women with breast cancer (invasive breast cancer or ductal carcinoma in situ) subsequently diagnosed after recruitment to the study. All saliva samples were collected before January 2014.

The PROCAS study was approved by the North Manchester Research Ethics Committee (Ref. 09/H1008/81).

Saliva samples were collected to extract DNA for SNP genotyping. DNA samples were stored at − 20 °C. The 18 SNPs (Supplemental Table 1) were genotyped as previously described [14, 15], blinded to whether the patient had developed breast cancer, by a custom designed Sequenom MassARRAY iPLEX assay or TaqMan® SNP Genotyping Assay. Per-allele odds ratios (OR) were derived from published OR and allele frequency as described previously by normalising around a relative risk of 1.0 [14, 15]. Briefly, the PRSs were calculated by multiplying the per-allele OR for each SNP (when a single SNP failed the woman was given an arbitrary score of 1.0 for that SNP). The PRS was used in further statistical analyses. Mammographic density was estimated by two readers using visual analogue scales, as previously described [10]. Density was adjusted for BMI and age and reported as a ‘density residual’ (DR) and was also expressed as a predictive odds ratio [10]. Women with bilateral breast cancer on prevalent study screen or with breast implants had no assessable VAS score and were given a nominal OR of 1.0 for density residual in the combined analysis of all three measures.

TC v.6 10-year risk was calculated based on the questionnaires completed by PROCAS participants at study entry. The questionnaire included data on age, age at first full-term pregnancy, BMI (from height/weight), number of affected first and second-degree relatives, history of previous breast biopsy, parity and ethnicity. We have previously demonstrated very low correlation between SNP18 and TC, and SNP18 with DR [15]. Thus, no adjustments were made for SNP18 PRS when incorporated 0 into the 10-year risk estimate as SNP18 was almost completely calibrated (observed to expected odds ratio for SNP18 was 0.99, 95% CI 0.70–1.26).

Clinical endpoints examined in the present study were: breast cancer characteristics obtained from histopathology reports: invasive tumour vs ductal carcinoma in situ (DCIS), invasive tumour grade, stage and estrogen receptor (ER) and human epidermal growth factor receptor 2 (HER2) status. Further, breast cancer cases were subdivided into a category of extremely good prognosis (EGP) that was not previously defined but was used to capture those cancers that would either have been unlikely to have caused a patient death or may never have presented clinically i.e. invasive tumours that were both stage 1 and grade 1 occurring in those > 60 years or intermediate grade DCIS > 55 years or low-grade DCIS at any age.

10-year breast cancer risk was stratified into four main groups: < 2%; 2–3.49%; 3.5–4.99% and ≥ 5% risk as previously [15], the latter group combining UK National Institute for Clinical and Care Excellence (NICE) defined high and moderate risk groups for which additional screening and chemoprevention are recommended [16]. Additionally, the moderate/high-risk group was split further as per NICE guidelines into moderate (5%-7.99%) and high-risk (8%+), and an additional very low risk group with a < 1.5% 10-year risk (the mean 10-year population risk at age 40 years, where screening is not recommended in the UK). Prospective follow-up was censored at date of BC diagnosis date of death (from NHS Hospital Episode Statistics data) or date cancer databases were last checked via the cancer registries (30/09/2017).

Incident breast cancers were those occurring after DNA sample collection. DR and TC were also studied in the wider PROCAS cohort.

### Statistical methods

Two sets of cases were analysed: (1) all breast cancers diagnosed subsequent to completion of the questionnaire and (2) those diagnosed subsequent to saliva collection for analysis of DNA.

Odds ratios per interquartile range (IQR-OR) with 95% Wald confidence intervals were calculated by logistic regression, using the natural logarithm for each of the risk factors assessed (TC, DR, SNP18), to examine the relationship between cases and controls for the different pathological types (invasive tumour grade, DCIS, stage 2+, ER+, ER−, HER2+ and all breast cancer).

In the prospective analysis following saliva collection, relative risks were estimated with 95% confidence intervals from an exact Poisson method. Expected risk from the model was obtained by converting expected incidence to cumulative hazard i.e., cumulative hazard H = − log (1 − incidence) and summing.

## Results

### DNA cohort

Of the 9899 women with saliva DNA, 537 were excluded as having previously been diagnosed with breast cancer. Of the remaining 9362 women unaffected at study entry, 270 were diagnosed at the prevalent mammogram in PROCAS with 30 being diagnosed after their prevalent study mammogram but before saliva DNA collection. This left 9062 who had not been diagnosed with breast cancer before DNA collection. In these 9062 women there were 56057.6 women years of follow-up (mean 6.19 years, IQR 5.46–6.96), indicating that most women had two further screening mammograms within the study period. Two were lost to follow-up and 167 had died at the time of analysis, 6 from an incident breast cancer.

### Incident breast cancers

**Fig. 1:**
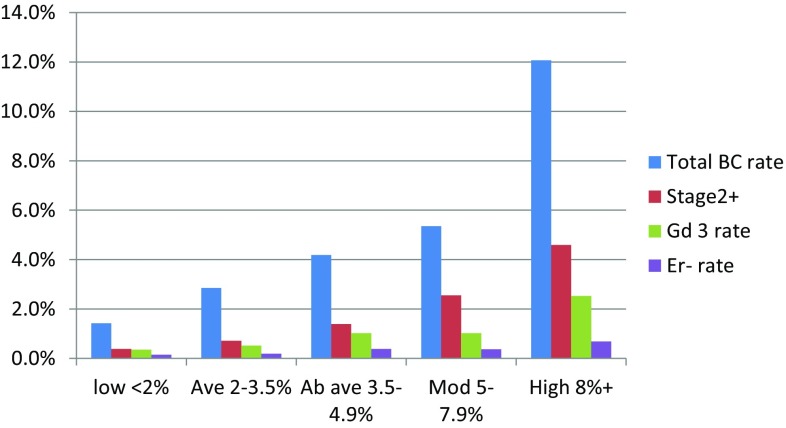
10-year actual prospective breast cancer rates by combined TC-DR-SNP18 group excluding prevalent cancers at first screen

There were 195 incident breast cancers (26 (13.3%) of which were DCIS) with 195.5 expected by a combined TC/DR/SNP18 risk calculation (O:E ratio = 0.997). There were 184.3 expected by TC assessment alone (Table 1). The 10-year rates of breast cancer in each group were accurately predicted (Fig. 1), with point estimates within the expected range, even in the very low-risk group (< 1.5%). The combined model performed well in predicting subsequent breast cancer overall (IQ-OR 2.25 (1.89–2.68)) and performed particularly well in predicting higher stage stage 2+ IQ-OR = 2.69 (2.02–3.60) and ER + BCs (ER + IQ-OR = 2.36 (1.93–2.89)).

**Table 1:** Women unaffected by breast cancer at entry with DNA collected by combined 10-year risk groups

10-year risk group	< 1.5%^a^	< 2%	2–3.49%	3.5–4.9%	5–7.9%	8% +	Total
Total in group	1596	3003	3038	1587	1129	605	9362
BC before sampling	23	59	87	76	45	33	300
Number in prospective group	1573	2944	2951	1511	1084	572	9062
% in Group	17.36%	32.49%	32.56%	16.67%	11.96%	6.31%	100.00%
Prospective BC number	10	26	52	39	36	42	195
Prospective DCIS	2 (20%)	4 (15.4%)	8 (15.4%)	5 (12.8%)	5 (13.9%)	4 (9.5%)	26 (13.3%)
Stage 2+	2 (20%)	8 (30.8%)	13 (25%)	13 (33.3%)	17 (47.2%)	16 (38.1%)	68 (34.9%)
% with prospective BC/total	0.64%	0.88%	1.76%	2.58%	3.32%	7.34%	2.15%
% of all stage 2+ cancers	2.9%	10.3%	19.1%	19.1%	23.5%	25%	
Years of follow-up	9845.9	18361.8	18263.7	9304.1	6645.8	3482.1	56057.6
Expected BC by TCDRSNP	11.17	26.14	48.61	38.52	40.89	41.36	195.51
Expected BC by TC	21.23	42.89	54.39	34.04	30.44	22.51	184.27
Rate 10-year	1.02%	1.42%	2.85%	4.19%	5.42%	12.06%	3.48%
Rate 10-year Stage 2+	0.2%	0.43%	0.71%	1.39%	2.55%	4.59%	
OR of stage 2+ incidence	Ref	2.15	3.55	6.95	12.75	22.95	

^a^The 1.5% risk group includes women also in the < 2% group to assess which is the better low-risk threshold

### All with DNA including prevalent

**Fig. 2:**
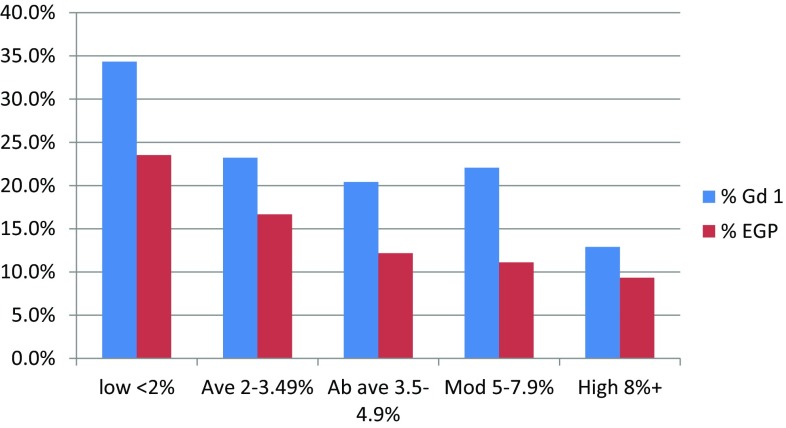
Proportion of cancers identified in each TC/DR/SNP18 group that are grade 1 or extremely good prognosis (EGP)

Utilising all the available tumour pathology from prevalent (only contralateral breast density was used as previously [14, 15]) and incident cancers from 9362 women combined, TC, DR and SNP18 were individually predictive of breast cancer as a whole, as well as most pathological subtypes. However, the strength of prediction of each individual risk factor (TC, DR and SNP18) varied considerably for several tumour subtypes (Table 2). Whilst DR was more predictive of interval cancers and stage 2+ cancers on first screen it was less predictive than SNP18 of incident stage 2+ cancers. DR was particularly predictive of HER2+ breast cancer with an interquartile (IQR) OR of 1.96 (95% CI 1.28–2.99). The only category that DR was not predictive of was ER negative with an IQR OR of 1.13 (95% CI 0.73–1.76), similar to that for SNP18 OR 1.15 (95% CI 0.74–1.80); however, TC was modestly predictive for ER-negative cancers: IQR OR 1.39 (95% CI 0.96–2.03, *P* = 0.09). Furthermore, DR was more predictive for grade 1 and EGP breast cancers than either TC or SNP18 (Table 2; Fig. 2).

**Table 2:** Logistic regression for prediction of pathology parameters compared to no breast cancer using inter-quartile odds ratios (significant differences in bold type)

	Tyrer-Cuzick				Density residual			SNP18		
	Number	OR per IQR	95% CI	LR ${\chi ^2}$	OR per IQR	95% CI	LR ${\chi ^2}$	OR per IQR	95% CI	LR ${\chi ^2}$
No BC	8867	1.00	Ref		1.00	Ref		1.00	Ref	
BC	495	**1.29**	**(1.16–1.43)**	**21.95**	**1.58**	**(1.41–1.78)**	**56.87**	**1.55**	**(1.37–1.75)**	**49.92**
Incident stage 2+	68	0.95	(0.71–1.27)	0.13	**1.59**	**(1.17–2.16)**	**8.43**	**1.79**	**(1.30–2.46)**	**12.65**
All stage 2+	136	**1.32**	**(1.09–1.60)**	**7.60**	**1.84**	**(1.49–2.29)**	**29.77**	**1.72**	**(1.37–2.15)**	**21.62**
Incident interval	91	**1.56**	**(1.24–1.96)**	**13.60**	**1.73**	**(1.33–2.25)**	**15.94**	**1.53**	**(1.16–2.02)**	**8.98**
Grade 1	94	1.06	(0.83–1.35)	0.19	**1.55**	**(1.20–2.02)**	**10.52**	1.21	(0.92–1.58)	1.78
EGP	78	0.95	(0.72–1.25)	0.14	**1.67**	**(1.26–2.22)**	**11.97**	**1.36**	**(1.00–1.83)**	**3.94**
Grade 3	102	**1.35**	**(1.08–1.69)**	**6.77**	**1.42**	**(1.10–1.83)**	**7.15**	**1.37**	**(1.06–1.79)**	**5.62**
DCIS	90	**1.27**	**(1.00-1.62)**	**3.69**	**1.59**	**(1.22–2.08)**	**11.34**	**1.49**	**(1.13–1.96)**	**7.77**
ER negative	35	1.39	(0.96–2.03)	2.85	1.13	(0.73–1.76)	0.29	1.15	(0.74–1.80)	0.37
ER positive	358	**1.26**	**(1.12–1.43)**	**13.49**	**1.63**	**(1.42–1.87)**	**47.48**	**1.62**	**(1.41–1.87)**	**44.77**
HER2+	34	1.42	(0.97–2.08)	3.12	**1.96**	**(1.28–2.99)**	**9.19**	1.25	(0.79–1.96)	0.91

In view of the potential benefits of lower prediction probabilities when the cancer is grade 1 or EGP where there is a great risk of overtreatment we looked at the full TC/DR/SNP18 prediction model for such cancers (Table 3). There was a higher proportion of grade 1 breast cancers as a proportion of all invasive breast cancers in the low-risk group: 23/67 (34.3%) compared to 15/66 (22.7%) in the moderate and 8/58 (13.8%) in the high risk group (*P* = 0.007; Table 3). The proportion of EGP as defined in the methods was also significantly higher at 20/85 (23.5%) in the low-risk group compared to the moderate/high-risk group at 16/155 (10.3%); *P* = 0.008 (Table 1).

**Table 3:** Breast cancer pathology by combined TC/DR/SNP18 risk groups

TC/MD/SNP18 10-year risk%	Low < 2%	Average 2–3.49%	Above average 3.5–4.9%	Moderate/high ≥ 5%	Total
Number of women	3003	3038	1587	1734	9362
Invasive BC	67	110	98	124	399
Total BC	85	140	115	155	495
Proportion with BC	2.83%	4.58%	7.25%	8.9%	5.29%
Invasive grade 1	23	26	20	23	92
% Gd1 of invasive in group	34.33%	23.64%	20.41%	18.55**%**	23.06%
Adjusted Gd1 10-year incidence rate^a^	0.38%	0.53%	0.73%	1.14%	0.65%
Invasive grade 2	23	57	46	73	199
% Gd2 in group	34.33%	51.82%	46.94%	58.87%	49.87%
Adjusted Gd2 10-year incidence rate^a^	0.38%	1.16%	1.68%	3.63%	1.40%
Invasive grade 3	20	27	29	26	102
%Gd3 in group	29.85%	24.54%	29.59%	20.97%	25.56%
Adjusted Gd3 10-year rate^a^	0.33%	0.55%	1.06%	1.29%	0.72%
Grade unknown	1	0	3	2	6
ER + invasive BC	58	98	86	116	358
ER− invasive BC	9	9	10	7	35
ER unknown	1	3	0	1	5
DCIS	18	27	17	29	91
%DCIS	21.18%	18.84%	14.78%	18.82%	17.94%
Cancer type unknown	0	3	0	2	5
EGP	20	23	14	16	73
%EGP in group	23.53%	16.55%	12.17%	10.32%	14.75%
Rate per 1000, invasive ER− per 10-years	0.16%	0.19%	0.38%	0.37%	0.26%
10-year rate, ER+	1.26%	2.66%	3.81%	7.23%	3.22%
10-year rate, all BC	1.42%	2.85%	4.19%	7.6%	3.48%

9/24 (37.5%) of the invasive cancers in those at < 1.5% risk were grade 1 compared to 8/58 (13.8%) in those at 8%+ risk (p = 0.02). However 3/33 (9%) of total cancers in those at < 1.5% risk were invasive ER− compared to 4/82 (4.9%) for those at 8%+ risk

^a^Incidence rates are adjusted to actual incidence from time of saliva collection

### Incident breast cancers

Overall, the addition of SNP18 to TC/DR increased the proportion of women in the low-risk group after saliva collection (< 2% 10-year risk) from 26.8% (2426/9062) to 32.5% (2944/9062). Despite this increase in number, there were proportionately fewer incident BCs in the group defined by TC, DR and PRS combined vs TC/DR alone [0.88% (26/2944) vs 1.03% (25/2426)] thus the ratio to the overall mean population breast cancer incidence using TC/DR/SNP18 was improved to the lower figure of 0.40 (95% CI 0.26–0.59) from 0.54 (95% CI 0.35–0.82) using TC/DR. Similarly, the addition of SNP18 to TC/DR increased the proportion of women in the moderate/high-risk group (≥ 5% 10-year risk) from 16.4% (1487/9062) to 18.3% (1656/9062) and the proportion of total breast cancers in these groups also increased from 65/195 (33.3%) using TC/DR to 78/195 (40%) using TC/DR/SNP18 and proportionately more of these women developed BC 4.37% (65/1487) versus 4.71% (78/1656) This results in comparable ratios of population to breast cancers of 2.03 (95% CI 1.52–2.69) and 2.19 (95% CI 1.67–2.85) respectively. Therefore, adding SNP18 to TC/DR led to greater separation of the population into high and low-risk groups, and potentially for stage 2+. Only 8/2944 (0.27%) women in the low-risk group developed an incident stage 2+ BC on combined TC/DR/SNP18 analysis compared to 33/1656 (2.11%) in the mod/high-risk group, a 7.8 fold difference. Using just TC/DR there were 6/2426 (0.25%) in low risk compared to 26/1487 (1.75%) in the moderate/high-risk group a sevenfold difference. Overall, in the incident population after saliva collection of 9062 women just over half (4600/9062-50.7%) could be divided into either low or moderate/high risk using TC/DR/SNP18 compared to 3913 (43.1%) using TC/DR alone. This resulted in better prediction ratios overall, with an OR from low to moderate/high of 5.44 (95% CI 3.52–8.61) with TC/DR/SNP18 compared to 4.36 (95% CI 2.77–7.02) using TC/DR alone.

The predictive value for ER-negative breast cancer with TC/DR/SNP18 was weaker although numbers were small. Overall, only 35/405 (8.6%) invasive breast cancers were ER−, with 32 (7.9%) being triple negative.

Of all cancers diagnosed in the TC/DR/SNP18 group, 136/495 (27.5%) were stage 2 or above. 48 (35%) of these cases were in the high/moderate-risk group (33/68–48.5% of incident BC) compared with only 16 (11.7%) in the low-risk group (8–11.7% of incident BC); *P* < 0.0001. The proportion of stage 2+ cancers in the low risk group by TC/DR/SNP18 was 16/85 (18.8%) compared to 48/155 (30.9%) in the moderate/high-risk group (*P* = 0.05). The rate of stage 2+ cancer post-prevalent was 7.41 times higher (95% CI 3.57 to 17.28) in the moderate/high-risk group (3.26 per 1000, 95% CI 2.32–4.58) compared to the low-risk group at 0.44 (95% CI 0.22–0.87); *P* < 0.0001. Of the 167 women who have died in the whole DNA/PROCAS cohort, thirteen died as a result of a breast cancer diagnosis since entering PROCAS. Nine of the thirteen deaths (69%) were in those with a 10-year risk > 3.5% and nine of the thirteen were stage 2+ at diagnosis.

The TC/DR/SNP18 score may be useful to define a group of women attending for their first population screening mammogram (aged 46–52 years in the UK) who do not require screening for a 10-year period. For example, the group aged 46–52 years at < 2% 10-year risk in PROCAS had a mean 10-year incidence of only 1.4% (5 of 547 developed breast cancer after 3383.27 years of follow up) and for the 262 women at < 1.5% 10-year risk there was only 1 breast cancer in 1648.43 years giving a rate of 0.6 per 1000 women in 10 years.

### Full PROCAS dataset

Using the full PROCAS dataset of 57,902 women unaffected at entry, the DR was not predictive of ER− cancers. The median DR was 0.99 (IQR 0.84–1.19) for ER− tumours and 1.04 (0.87–1.26) for ER + cancers; the IQR OR compared to no cancer was 1.58 (95% CI 1.46–1.70) for ER+ and 1.31 (95% CI 1.06–1.31) for ER–. Similarly, TC 10-year risk median IQR was also less predictive of ER− at 2.84 (2.18–3.59) for ER− versus 2.91 (2.27–4.07) for ER+. This gave an IQR OR of 1.19 (95% CI 0.99–1.43) for ER− and 1.39 (95% CI 1.30–1.48) for ER+. In contrast using the full PROCAS dataset, DR was highly predictive of HER2+ breast cancer with an IQR OR of 1.72 (95% CI 1.39–2.15) compared to 1.54 (95% CI 1.44–1.66) for all breast cancer.

## Discussion

We have shown that combining TC, DR and SNP18 improves the accuracy of breast cancer risk stratification over each factor independently and helps predict which women are more likely to develop better and worse prognosis cancers. Previously, we demonstrated that there was a higher proportion of cancers that are interval and stage 2+ in women at high/moderate-risk than for those at low risk [15]. The present study adds a further 1.3 years of prospective follow-up, 30 more incident cancers and details of the pathology of the breast cancers diagnosed. We are unable to find any previous report of a prospective risk stratification study that has shown differences in grade and stage of breast cancers in relation to risk. The results suggest that in a 3-yearly mammography screening programme more frequent imaging, potentially including newer techniques such automated breast ultrasound (ABUS), contrast-enhanced mammography or MRI may be indicated to down-stage breast cancers in the moderate/high-risk groups. Although high stage invasive cancers still occur in the low risk group this has to be balanced against the higher proportion of extremely good prognosis cancers, which is almost double that in the high/moderate risk group (23.5% vs 10.1%). A case could be made to assess women at screening entry at around 46–50 years of age, and those at a 10 years risk of < 2% or perhaps < 1.5% could be counselled that screening is not indicated now and they should be reassessed in a further 10-years. In those countries with 2-yearly screening programmes reduction in frequency to 3-yearly in the 33% at < 2% risk seems a reasonable option in order to offset the cost of the extra screening suggested in those at above average risk. Although the cost of a SNP PRS commercially is relatively high the current cost of an Illumina onco-array which allows testing of over 300 potential breast cancer SNPs is currently around $70USD per person and with saliva collection, DNA extraction and analysis a $100USD cost is feasible and would only need to be undertaken once.

The high proportion of stage 2+ and interval cancers in women with high mammographic density is likely to be due in part to ‘masking’ of smaller cancers in areas of dense fibroglandular tissue on mammography. This does not, however, explain the overall better prediction of post-prevalent stage 2+ cancers using SNP18.

Before any such change to screening intervals is introduced the effectiveness of this change could be enhanced by better identification of the worse prognosis ER− breast cancers. Although TC risk does to some extent predict these cancers they are less effective than for ER+ cancers. SNP18 has little predictive value as would be expected, as the majority of individual SNPs are associated with ER+ tumours [17], with only three predicting ER− cancer risk which are also predictive for breast cancer in women with *BRCA1* pathogenic variants, where the cancers are predominantly ER− [6]. The recent identification of ten new SNPs for ER− disease [18] alongside ten that were already discovered may provide a SNP20 for ER− breast cancer. A SNP20 PRS for ER− breast cancer would likely provide a more accurate prediction of more lethal ER− disease along with TC and density. Assuming 10–15% of breast cancers in the age range 46–73 years are ER—an acceptably low rate of both total risk of breast cancer of < 2% or perhaps < 1.5%, alongside a rate of ER− of < 0.3% could constitute a reasonable threshold to delay further screening until reassessment at 10 years, unless risk factors change in the interim.

We have identified a strong link between mammographic density and HER2+ breast cancer and this has also been highlighted recently [19].

There are limitations of the present study. We have used a definition for extremely good prognosis breast cancer that is likely to contain the great majority of potential over-diagnosis. However, grade 1 stage 1 breast cancers are still capable of causing breast cancer deaths and if women are removed from screening they are likely to become higher stage before becoming symptomatic. Equally, intermediate grade DCIS could become at least a grade 2 invasive breast cancer if untreated, with the possibility of a later breast cancer-related death. There was some missing data from the present study and two women were lost to follow-up and 18/407 (4.4%) invasive breast cancers had missing grade and ER status. Strengths include the large number of fully genotyped women in the prospective analysis with mammographic density information.

In conclusion, in this study, we report that the risk groups not only define incidence rates but also the pathology and prognosis of the breast cancers that develop in them, with important implications for screening and preventive strategies. The current study confirms the added accuracy of risk prediction using a combined TC/DR/SNP18 approach and that it can define a sizeable group (32.5%) of women who have a low (< 2%) 10-year risk. Furthermore, cancers identified in this group are significantly more likely to have an extremely good prognosis [17]. This work provides important evidence for a risk-stratified approach to breast cancer screening with an assessment at first mammogram around age 45–50 where extra screening in the high-risk group to reduce the risk of stage 2+ cancers could be offset by reducing or eliminating screening in the larger low-risk group, where the benefits of screening may be outweighed by false-positive screens and the potential for over-diagnosis and over-treatment.

## Electronic supplementary material

Below is the link to the electronic supplementary material.

Supplementary material 1 (DOC 49 KB)

## References

[1] Eccles SA, Aboagye EO, Ali S (2013). Critical research gaps and translational priorities for the successful prevention and treatment of breast cancer. Breast Cancer Res.

[2] Michailidou K, Beesley J, Lindstrom S (2015). Genome-wide association analysis of more than 120,000 individuals identifies 15 new susceptibility loci for breast cancer. Nat Genet.

[3] Michailidou K, Lindström S, Dennis J (2017). Association analysis identifies 65 new breast cancer risk loci. Nature.

[4] Kapoor N, Curcio LD, Blakemore CA, Bremner AK, McFarland RE, West JG, Banks KC (2015). Multigene panel testing detects equal rates of pathogenic BRCA1/2 mutations and has a higher diagnostic yield compared to limited BRCA1/2 analysis alone in patients at risk for hereditary breast cancer. Ann Surg Oncol.

[5] Thompson ER, Rowley SM, Li N, McInerny S, Devereux L, Wong-Brown MW, Trainer AH, Mitchell G, Scott RJ, James PA, Campbell IG (2016). Panel testing for familial breast cancer: calibrating the tension between research and clinical care. J Clin Oncol.

[6] Evans DG, Brentnall A, Byers H, Harkness E, Stavrinos P, Howell A, Newman WG, Cuzick J, FH-risk study Group (2017). The impact of a panel of 18 SNPs on breast cancer risk in women attending a UK familial screening clinic: a case-control study. J Med Genet.

[7] Tyrer J, Duffy SW, Cuzick J (2004). A breast cancer prediction model incorporating familial and personal risk factors. Stat Med.

[8] Gail MH, Brinton LA, Byar DP, Corle DK, Green SB, Schairer C, Mulvihill JJ (1989). Projecting individualized probabilities of developing breast cancer for white females who are being examined annually. J Natl Cancer Inst.

[9] Warwick J, Birke H, Stone J, Warren RM, Pinney E, Brentnall AR, Duffy SW, Howell A, Cuzick J (2014). Mammographic breast density refines Tyrer-Cuzick estimates of breast cancer risk in high-risk women: findings from the placebo arm of the International Breast Cancer Intervention Study I. Breast Cancer Res.

[10] Brentnall AR, Harkness EF, Astley SM (2015). Mammographic density adds accuracy to both the Tyrer-Cuzick and Gail breast cancer risk models in a prospective UK screening cohort. Breast Cancer Res.

[11] Evans DG, Warwick J, Astley SM, Stavrinos P, Sahin S, Ingham S, McBurney H, Eckersley B, Harvie M, Wilson M, Beetles U, Warren R, Hufton A, Sergeant JC, Newman WG, Buchan I, Cuzick J, Howell A (2012). Assessing individual breast cancer risk within the U.K. National Health Service Breast Screening Program: a new paradigm for cancer prevention. Cancer Prev Res (Phila).

[12] Dite GS, MacInnis RJ, Bickerstaffe A, Dowty JG, Allman R, Apicella C, Milne RL, Tsimiklis H, Phillips KA, Giles GG, Terry MB, Southey MC, Hopper JL (2016). Breast cancer risk prediction using clinical models and 77 independent risk-associated SNPs for women aged under 50 years: Australian Breast Cancer Family Registry. Cancer Epidemiol Biomark Prev.

[13] Vachon CM, Pankratz VS, Scott CG (2015). The contributions of breast density and common genetic variation to breast cancer risk. J Natl Cancer Inst.

[14] Evans DG, Astley S, Stavrinos P, Harkness E, Donnelly LS, Dawe S, Jacob I, Harvie M, Cuzick J, Brentnall A, Wilson M, Harrison F, Payne K, Howell A (2016). Improvement in risk prediction, early detection and prevention of breast cancer in the NHS Breast Screening Programme and family history clinics: a dual cohort study.

[15] van Veen E, Brentnall AR, Byers H (2018). Improving classical breast cancer risk prediction with single nucleotide polymorphisms and mammographic density. JAMA Oncol.

[16] McIntosh A, Evans DG et al (2017) Clinical guidelines and evidence review for the classification and care of women at risk of familial breast cancer. London: National Collaborating Centre for Primary Care/University of Sheffield. NICE guideline CG014, 2004 (updated 2006 CG41, 2013/2017 CG184). http://www.nice.org.uk Accessed 17 Jun 2018

[17] Turnbull C, Ahmed S, Morrison J (2010). Genome-wide association study identifies five new breast cancer susceptibility loci. Nat Genet.

[18] Milne RL, Kuchenbaecker KB, Michailidou K (2017). Identification of ten variants associated with risk of estrogen-receptor-negative breast cancer. Nat Genet.

[19] Edwards BL, Atkins KA, Stukenborg GJ, Novicoff WM, Larson KN, Cohn WF, Harvey JA, Schroen AT (2017). The association of mammographic density and molecular breast cancer subtype. Cancer Epidemiol Biomark Prev.

