# Supplementary material for: Breast cancer pathology and stage are better predicted by risk stratification models that include mammographic density and common genetic variants
Source: Breast Cancer Res Treat. 2019 Apr 2;176(1):141–8. doi: 10.1007/s10549-019-05210-2 (PMC6548748; doi:10.1007/s10549-019-05210-2)
Supplement: Supplementary file 1 — Supplementary material 1 (DOC 49 KB) [file 10549_2019_5210_MOESM1_ESM.doc]

**Supplementary Table 1:** 18 SNPs used in this study.

| SNP | Gene/locus | Risk allele | RAF | OR | OR  0 risk alleles | OR  1 risk allele | OR  2 risk alleles |
| --- | --- | --- | --- | --- | --- | --- | --- |
| rs614367 | 11q13 | T | 0.15 | 1.21 | 0.94 | 1.14 | 1.38 |
| rs704010 | *ZMIZ1* | A | 0.39 | 1.08 | 0.94 | 1.02 | 1.10 |
| rs713588 | 10q | A | 0.60 | 0.99 | 1.01 | 1.00 | 0.99 |
| rs889312 | *MAP3K* | C | 0.28 | 1.13 | 0.94 | 1.05 | 1.17 |
| rs909116 | *LSP1* | T | 0.53 | 1.07 | 0.93 | 0.99 | 1.06 |
| rs1011970 | *CDKN2A* | T | 0.17 | 1.05 | 0.98 | 1.03 | 1.08 |
| rs1156287 | *COX11* | A | 0.71 | 1.07 | 0.91 | 0.97 | 1.04 |
| rs1562430 | 8q24 | G | 0.42 | 0.90 | 1.09 | 0.98 | 0.88 |
| rs2981579 | *FGFR2* | T | 0.42 | 1.27 | 0.81 | 1.03 | 1.30 |
| rs3757318 | *ESR1* | A | 0.07 | 1.16 | 0.98 | 1.13 | 1.32 |
| rs3803662 | *TOX3* | T | 0.26 | 1.23 | 0.89 | 1.10 | 1.36 |
| rs4973768 | *SLC4A7* | T | 0.47 | 1.09 | 0.92 | 1.00 | 1.10 |
| rs8009944 | *RAD51L1* | A | 0.75 | 0.96 | 1.06 | 1.02 | 0.98 |
| rs9790879 | 5p12 | C | 0.40 | 1.09 | 0.94 | 1.01 | 1.10 |
| rs10931936 | *CASP8* | C | 0.74 | 0.96 | 1.06 | 1.02 | 0.98 |
| rs10995190 | *ZNF365* | G | 0.85 | 1.16 | 0.77 | 0.89 | 1.04 |
| rs11249433 | *NOTCH* | C | 0.42 | 1.09 | 0.93 | 1.01 | 1.10 |
| rs13387042 | 2q | A | 0.49 | 1.14 | 0.88 | 1.00 | 1.13 |

RAF: risk allele frequency; OR: odds ratio. Risk allele frequencies and odds ratios sought from the iCOGS database
